# Change in Alcohol Consumption and Binge Drinking in University Students During the Early COVID-19 Pandemic

**DOI:** 10.3389/fpubh.2022.854350

**Published:** 2022-04-27

**Authors:** Annina Zysset, Thomas Volken, Simone Amendola, Agnes von Wyl, Julia Dratva

**Affiliations:** ^1^Department of Health Science, Institute of Public Health, Zurich University of Applied Sciences, Winterthur, Switzerland; ^2^Department of Dynamic and Clinical Psychology, and Health Studies, Faculty of Medicine and Psychology, Sapienza University of Rome, Rome, Italy; ^3^Departement of Applied Psychology, Psychological Institute, Zurich University of Applied Sciences, Zurich, Switzerland; ^4^University of Basel, Basel, Switzerland

**Keywords:** risky health behavior, alcohol, binge drinking, anxiety, students, emerging adulthood, COVID-19, lockdown

## Abstract

**Objectives:**

Young adults have been overly affected by the containment measures against COVID-19 and, consequently, worsening in mental health and change in health behavior have been reported. Because the life phase of emerging adulthood is crucial for developing health behaviors, this study aims to examine increase in alcohol consumption, single and multiple binge drinking, and associated factors in students during lockdown and post-lockdown periods.

**Methods:**

A prospective open cohort study design with nine survey time points between April 2020 and June 2021 was conducted. The present study uses pooled data from the first survey T0 (3 April to 14 April) and follow-ups at T1 (30 April to 11 May 2020) and T2 (28 May to 8 June 2020). Students from all faculties of the Zurich University of Applied Sciences (ZHAW) (*N* = 12'431) were invited. Of the 1,300 students who participated at baseline and in at least one follow-up, 1,278 (98.3%) completed the questionnaires, final net sample size was 947. Generalized Estimating Equations (GEE) models were used to investigate the factors associated with increases in alcohol consumption based on number of occasions/last 30 days; drinks/week, and binge drinking at T0, and respective changes at T1 and T2 (increases, decreases, no change).

**Results:**

Overall, 20% of Swiss university students reported an increased alcohol consumption and 26% engaged in binge drinking. Number of drinks at baseline was associated with a higher probability of increased alcohol consumption, as well as engaging in single and multiple binge drinking events. Higher anxiety scores were associated with a higher probability to increase the alcohol consumption and engaging at least once in binge drinking. Additional factors associated with any binge drinking were male gender, younger age and not living with parents. Higher perceived social support was only associated with engaging in heavy binge drinking.

**Conclusions:**

A substantial number of students developed a more risky health behavior regarding alcohol consumption. It is important to identify at risk students and design target prevention including factors such as age, gender and social norms. Further, health behavior and determinants of health behaviors of students should be carefully monitored during the further course of the pandemic.

## Introduction

Public health measures taken in response to the COVID-19 pandemic substantially affected daily social and personal life of young adults, and educational settings (1). The challenges for young adults during the pandemic are voiced by Richter et al. (2): “It is safe to assume that many young people are experiencing significant hardship during this time.” Indeed, social contacts and mobility were restricted due to the national containment measures such as social distancing and closure of schools and universities. The daily structure of young adults changed drastically. Previous studies have shown that university students struggled with online classes and lack of contact with their fellow students (3, 4). Further, an increase in mental health symptoms in university students during the course of the pandemic has been observed (5–11). Moreover, change in health behaviors such as physical activity, nutrition and substance consumption in university students was reported (5, 6, 12–16).

Results on alcohol consumption during the lockdown are inconsistent. The assumption that university students would increase their consumption of alcohol during the lockdown, because of the stressful situation and lack of social control, was supported only by some of the studies collecting data during the pandemic (14, 15, 17). However, despite a part of university students increasing their alcohol consumption, others reduced their consumption. For example, Bann et al. examined change in alcohol consumption before and during the lockdown, in five representative British cohorts. In the youngest cohort, the 19-20-year-olds, 28% reported to have never consumed alcohol during the lockdown, and high risk alcohol consumption decreased from 33 to 13% (5). Similarly, college students in the USA reduced their quantity of alcoholic beverages from 11.5 to 9.9 drinks per week during the COVID-19 pandemic (16). However, another study from the USA found an increase in alcohol consumption in university students during the lockdown (14). Risk factors associated with an increased alcohol consumption were male gender and symptoms of depression and anxiety (5, 14), while greater perceived social support and returning to live with parents were associated with no increase in alcohol consumption (14, 16, 18). Those that perceived a high impact were more vulnerable to develop risky health behavior and adverse health outcomes (19, 20). These results indicate that the pandemic does not affect all young adults equally.

Psychological disorders and problematic substance consumption are related. On one hand, alcohol may be used as coping strategy to deal with symptoms of depression and anxiety disorders, and on the other hand, problematic alcohol consumption can increase the probability to develop a psychological disorder (21). This correlation between depression or anxiety disorders and increased alcohol consumption was also found during the pandemic (14). Alcohol abuse itself has damaging short- and long-term health effects for the individual (22, 23) and economic consequences at the individual and societal level (24). It is crucial to detect both, psychological symptoms and problematic alcohol consumption, at an early stage to intervene and recuse negative consequences, especially in young adults. The life phase of emerging adulthood is a vulnerable phase for the development and establishment of healthy and risky health behavior (25, 26), and the pandemic likely increased this vulnerability. While the pandemic most probably affected all young adults and university students, the impact on mental health and alcohol consumption may differ across countries.

Given differences in the epidemiological course of the pandemic, e.g., number of cases and mortality, and the containment measures implemented by country, one may assume a differential impact of the pandemic regarding factors associated with risky health behavior and individuals at risk of increasing risky health behavior in this age group. So far, only one other study has examined prevalence of alcohol consumption in Swiss university students in Switzerland, however, without analyzing predictors for alcohol consumption during the pandemic (8).

The overarching aim of the “HEalth in Students study during the Corona pandemic” (HES-C) was to (1) evaluate the **mental condition** of university students during the epidemic, (2) investigate changes in **health behavior** and (3) associated factors of both outcomes, such as pandemic related ones as well as individual factors. This article focusses on change in alcohol consumption and factors associated with an increase in alcohol consumption and binge drinking, corresponding to problematic alcohol consumption, during and after the first COVID-19 lockdown in Swiss university students.

## Methods

### Study Design and Study Sample

In HES-C, we employed a prospective open cohort study design with nine survey time points between 3^rd^ of April 2020 and 23^th^ of June 2021, each covering seven working days. The present study uses pooled data from the first survey T0 (3 April to 14 April 2020) and follow-ups at T1 (30 April to 11 May 2020) and T2 (28 May to 8 June 2020). The first survey T0, the baseline survey, took place during the first lockdown (16 March to 26 April 2020), while T1 and T2 took place after the lockdown. The Supplementary Figure 1 shows the corresponding stringency of containment measures in Switzerland, the epidemiological data incidence and deaths, as well as the survey time points. University students from all faculties of the ZHAW (*N* = 12'431) received a non-personalized email, via a central email distribution list containing all students, with information about the study and an online-link inviting them to fill in the online-survey. Of those invited 16.3% (T0, *n* = 2'023), 11.6% (T1, *n* = 1'436) and 9.7% (T2, *n* = 1'207) filled in the surveys. Participants had to actively provide their consent to participate in the study before filling in the online questionnaire. Anonymity was guaranteed at all times. The study is in accordance with the Declaration of Helsinki and was approved by both the local cantonal ethics committee (BASEC-Nr. Req-2020-00326) and the ZHAW data protection officer.

Of the 1,300 university students who participated in the baseline survey and in at least one follow-up survey, 1,278 (98.3%) completed the questionnaires in full. For the present study, we excluded university students who did not complete the respective surveys (*n* = 22), did not drink any alcohol at either survey (*n* = 194), did not provide information on alcohol consumption (*n* = 137) and university students who identified themselves as “other” gender (*n* = 4), resulting in a net sample size of 947.

### Outcomes and Measures

#### Outcomes

In our study, we assessed three outcomes related to alcohol consumption at T1 and T2 follow-ups: (*1) increased alcohol consumption in the past 30 days, (2) any binge drinking in the past 30 days (*≥ *5 drinks on one or more occasion), and (3) heavy binge drinking in the past 30 days (*≥ *5 drinks on two or more occasion)*. All outcomes were coded as binary response variables. Survey questions related to alcohol consumption were adopted from the European School Survey Project on Alcohol and other Drugs (ESPAD) (27, 28). Increased alcohol consumption was assessed using the following question: “Think back over the last 30 days. Has your alcohol consumption changed in the last 30 days?” (dichotomous response categories created: 0 = less alcohol/no change, 1 = more alcohol). Any binge drinking and heavy binge drinking were assessed by asking participants the following question: “Think back again over the last 30 days. How many times (if any) have you had five or more drinks on one occasion? (A “drink” is a glass/bottle/can of beer (ca 50 cl), a glass/bottle/can of cider (ca 50 cl), 2 glasses/bottles of alcopops (ca 50 cl), a glass of wine (ca. 15 cl), a glass of spirits (ca. 5 cl) or a mixed drink.” To capture episodes of any binge drinking and heavy binge drinking respectively, response categories (0, 1, 2, 3–5, 6–9, ≥10) were recoded to 0 = no binge drinking (response 0) and 1 = yes, binge drinking (response ≥1), and 0 = no (0–1) and 1 = yes (2–10) for heavy binge drinking.

#### Covariables

*Alcohol consumption* in the last 30 days was collected at baseline (T0, baseline alcohol consumption) and was assessed using survey questions from the European School Survey Project on Alcohol and other Drugs (ESPAD) (27, 28). Participants were asked two related questions: (1) “On how many occasions (if any) have you had any alcoholic beverages to drink during the last 30 days?” (response categories: never, on 1 day, on 2 days, on 3 days, once a week, twice a week, 3–4 times a week, (almost) every day) and (2) “Think back again over the last 30 days. When you were drinking alcohol, how many drinks on average did you have on each occasion?” (response categories: I do not drink alcohol, I did not drink alcohol during the last 30 days, 1 drink, 2 drinks, 3 drinks, 4 drinks, 5 drinks, 6 or more drinks). Total baseline alcohol consumption (total number of drinks) during the past 30 days was calculated by multiplying the average number of drinks per occasion (maximum fixed at 6 drinks) by the average number of days with alcohol consumption in the past 30 days (maximum fixed at 30).

##### Current Health Status

The participants were asked about their current state of health [adapted; (29)]. The question “What is your current state of health? Is it…” they could answer on a 5 point Likert scale (1 = very poor, 5 = very good).

##### Mental Health: Anxiety

Anxiety was measured with the General Anxiety Disorder-Scale-7 [GAD-7; (30)]. The GAD-7 is a self-assessment questionnaire that measures the level of perceived anxiety in the last 2 weeks. The questionnaire contains 7 items to be rated on a 4-point Likert scale (from “not at all” to “nearly every day”), resulting in a total score from 0 to 21. Total score can be interpreted as different levels of anxiety: minimal (0–4), mild (5–9), moderate (10–14) and severe (15–21). A review on pooled sensitivity and specificity values found cutoff scores 7–10 to have similarly moderate to high pooled estimates of sensitivity and specificity, [sensitivity: 0.74 (95% CI 0.61–0.84) to 0.85 (95% CI 0.73–0.92), specificity: 0.75 (95% CI 0.54–0.88) to 0.84 (95% CI 0.70–0.92)] (31).

##### Self-Efficacy

The General self-efficacy expectations Short scale [ASKU; (32)] is used to record individual expectations of competence in dealing with difficulties and obstacles in daily life. Therefore three statements had to be answered on a 5-point Likert scale (1 = doesn't apply at all, 5 = applies completely). Total score ranges from 3 to 15. The reliability of the ASKU lies between ω = 0.81 and 0.86, which corresponds to a sufficient reliability. The short version also has a sufficient reliability of α = 0.92 (32).

##### Resilience

Resilient coping was measured using the Brief Resilient Coping Scale [BRCS; (33)]. This instrument is based on four questions, which are answered on a 5-point Likert scale (1 = doesn't describe me at all, 5 = describes me very well). Total score ranges from 4 to 20 and allow the classification into the 3 categories: low resilience (4–13), medium resilience (14–16) and high resilience (17–20). The scale has a reliability of α = 0.69 and a predictive validity of α = 0.86 (33).

##### Social Support (Oslo)

The perceived availability of people whom individuals trust and who make them feel cared for, loved, respected and valued as a person was measured using the Oslo-3 Social Support Scale [Oslo-3; Kocalevent et al., (34)]. The total score ranges from 3 to 14 and can be divided into weak support (3–8), moderate support (9–11) and strong support (12–14). The scale has a low reliability of α = 0.50 and validity but considered acceptable (35).

##### Sociodemographic Variables

We collected the following sociodemographic information: age (year of birth), gender, socioeconomic status (SES) as measured by perceived parental social status at student age 16 years. (“Imagine a ladder with 10 rungs. Please mark on which rung you think your parents stood in relation to other people in Switzerland when you were 16 years old”) [MacArthur scale; (36)] and current living situation (I live (1) alone, (2) in a flat-sharing community, (3) with my partner, (4) with my parents/one parent or (5) other form of living). The living situation was dichotomized in living with parents and living autonomously.

### Statistical Analyses

Descriptive statistics (i.e., frequencies, percent, mean, and standard deviation) were applied to describe the characteristics of the sample. We used Student *t*-tests and Chi-square-tests to assess mean-level stability between measurement points.

Generalized Estimating Equations (GEE) models of the binomial family with logit link and robust standard errors, which adjust for repeated measures of the same subject, were employed to investigate the factors associated with increase in alcohol consumption and binge drinking. We estimated restricted models adjusting for time (follow-up 1 or 2) and the number of alcoholic drinks at baseline measurement in a first basic model. The full model adjusted additionally for age (centered at the mean value), gender, social status (centered at the mean value), living situation, health, anxiety (centered at the mean value), self-efficacy (centered at the mean value), resilience (centered at the mean value), social support (centered at the mean value), for the outcomes increased alcohol consumption, any binge drinking episode, and heavy binge drinking episodes in the past 30 days before the survey at either T1 or T2. We report odds ratios (OR) with corresponding 95% confidence intervals (95% CI), predictive margins (average predicted probability), and average marginal effects. Statistical significance was established at *P* < 0.05. We used Stata Version 15.1 (StataCorp, College Station, TX, USA) for statistical analyses.

#### Missing Data

Information on alcohol consumption, our primary outcome, was provided by 947 university students. Complete data for all variables used in the three different models were available for at least 97.4% of the cases. Social status had the highest number of missing values (2.1%), missing values for the remaining variables were in the range between 0.0 and 0.3%. We used the Stata mvpatterns and misschk commands to assess incomplete cases and to cross tabulate and plot all combinations of missing and non-missing values of the variables used in the respective models. Using visual inspection, we detected no systematic pattern in the missing data. For this reason and the low number of incomplete cases, we did not impute missing data (37) and included complete cases throughout all analyses.

## Results

Participant characteristics and descriptive statistics are shown in Table 1. Overall, the mean age of participants was 27.0 years (SD = 6.5) and 75.8% was female. Overall, 58.8% lived in their own households, the remaining ones lived with their parents. Participants' characteristics differed slightly between measurement points but were comparable (see Follow-up 1 and 2 in Table 1). The majority (87.5%) reported a good or very good health status. Mean anxiety score was 6.2 (SD = 4.2), corresponding to a medium anxiety level. Regarding the alcohol consumption, participants drank on average 13.5 alcoholic beverages (SD = 15.5) in the last 30 days. The majority reported no increase in alcohol consumption (80.2 %), while overall 19.8% increased their consumption (T1 24.9%, T2 14.1%). In our sample 26.9% engaged at least once in binge drinking and 6.5% engaged at least twice in binge drinking (T1 7.5%, T2 2 5.3%).

**Table 1:** Participant characteristics.

**Variable**	**Follow-up 1**	**Follow-up 2**	**Total**
	**% (*n*) or mean ± sd (*n*)**	**% (*n*) or mean ± sd (*n*)**	**% (*n*) or mean ± sd (*n*)**
**Gender**			
Women	75.5 (373)	76.2 (345)	75.8 (718)
Men	24.5 (121)	23.8 (108)	24.2 (229)
**Age**	26.9 ± 6.1 (494)	27.1 ± 6.5 (453)	27.0 ± 6.5 (947)
**Home**			
Lives in own household	59.8 (295)	57.7 (261)	58.8 (556)
Lives with parents	40.2 (198)	42.3 (191)	41.2 (389)
**Social status**	5.7 ± 1.5 (483)	5.7 ± 1.5 (444)	5.7 ± 1.5 (927)
**Health**			
Very poor – mediocre	13.8 (68)	11.0 (50)	12.5 (118)
Good – very good	86.2 (425)	89.0 (403)	87.5 (828)
**Anxiety (GAD score)**	6.2 ± 4.3 (494)	6.6 ± 4.1 (453)	6.2 ± 4.2 (947)
**Self-efficacy**	3.9 ± 0.6 (493)	3.9 ± 0.6 (452)	3.9 ± 0.6 (945)
**Resilience**	15.4 ± 2.2 (494)	15.3 ± 2.2 (453)	15.4 ± 2.2 (947)
**Social support**	10.6 ± 1.8 (493)	10.6 ± 1.7 (451)	10.6 ± 1.8 (944)
**Number of drinks at baseline**	14.2 ± 16.5 (494)	12.7 ± 15.3 (453)	13.5 ± 15.9 (947)
**Increased alcohol consumption**			
No	75.1 (371)	85.8 (389)	80.2 (760)
Yes	24.9 (121)	14.1 (64)	19.8 (187)
**Any binge drinking**			
No	71.9 (354)	74.3 (336)	73.1 (690)
Yes	28.1 (138)	25.7 (116)	26.9 (254)
**Heavy binge drinking**			
No	92.5 (455)	94.7 (428)	93.5 (883)
Yes	7.5 (37)	5.3 (24)	6.5 (61)

*N, number of observations; mean, arithmetic mean; sd, standard deviation*.

*Test for differences between follow-up 1 and 2 comprised Chi-2-test and Student t-test. No statistically significant differences were found*.

*Overall 559 (62.3%) university students participated in both follow-ups, 198 in follow-up 1 (20.9%), and 190 (16.8%) in follow-up 2*.

### Increased Alcohol Consumption

Reporting increased alcohol consumption in the past 30 days was associated with the number of drinks at baseline in the restricted model (OR = 1.04, 95% CI: 1.03–1.06), adjusting only for time (follow-up), and in the full model (OR = 1.04, 95% CI: 1.03–1.06), adjusting for all covariates (Table 2). At the second follow-up, university students were less likely to report increased alcohol consumption in the past 30 days as compared to the first follow-up (OR = 0.52, 95% CI: 0.38–0.70 and OR = 0.51, 95% CI: 0.37–0.70 in the restricted and full model respectively). With respect to covariates, higher anxiety scores (OR = 1.06, 95% CI: 1.01–1.11) were associated with increased alcohol consumption, and university students with higher resilience were less likely to report increased alcohol consumption (OR = 0.90, 95% CI: 0.82–0.99). Remaining covariates were not associated with increased alcohol consumption.

**Table 2:** Associations between time and participants' characteristics and increase in alcohol consumption and binge drinking^*^.

	**Increased alcohol consumption**		**Any binge drinking**		**Heavy binge drinking**	
	**(1)**		**(2)**		**(3)**	
**Variable**	**OR**	**95% CI**	**OR**	**95% CI**	**OR**	**95% CI**
*Follow-up 1 (Ref)*						
Follow-up 2	0.51^***^	[0.37, 0.70]	0.99	[0.93, 1.05]	0.92	[0.80, 1.06]
*Women (Ref)*						
Men	0.84	[0.52, 1.37]	2.24^***^	[1.40, 3.60]	1.98	[0.90, 4.36]
Age	1.02	[0.99, 1.05]	0.94^**^	[0.91, 0.98]	0.97	[0.90, 1.05]
Social status^*a*^	1.00	[0.88, 1.13]	0.97	[0.85, 1.11]	0.92	[0.72, 1.18]
Living situation						
*In own household (Ref)*						
In parent's household	0.72	[0.45, 1.16]	0.54^*^	[0.33, 0.87]	1.55	[0.60, 3.98]
Health						
*Very poor – mediocre (Ref)*						
Good – very good	0.94	[0.58, 1.54]	0.93	[0.71, 1.23]	0.80	[0.47, 1.34]
Anxiety score	1.06^*^	[1.01, 1.11]	1.06^*^	[1.01, 1.12]	0.99	[0.91, 1.08]
Number of drinks at baseline	1.04^***^	[1.03, 1.06]	1.08^***^	[1.05, 1.10]	1.08^***^	[1.06, 1.09]
Self-efficacy	0.96	[0.65, 1.42]	1.25	[0.79, 1.97]	1.74	[0.82, 3.70]
Resilience	0.90^*^	[0.82, 0.99]	1.00	[0.89, 1.12]	0.98	[0.80, 1.19]
Social support	1.03	[0.92, 1.16]	1.12	[0.99, 1.27]	1.28^*^	[1.00, 1.66]
*N*	925		922		922	

^*^Estimates from Generalized Estimating Equation (GEE) from the binomial family with logit link and robust standard errors. (1) Increased alcohol consumption in the past 30 days; (2) Any binge drinking past 30 days (≥ 5 drinks on one or more occasion); (3) Heavy binge drinking past 30 days (≥ 5 drinks on two or more occasion).

*OR, Odds Ratio; 95% CI 95%; Ref. = reference value, ^a^Social status = MacArthur scale (36)*.

* *p < 0.05*,

** *p < 0.01*,

*** *p < 0.001*.

**Figure 1:**
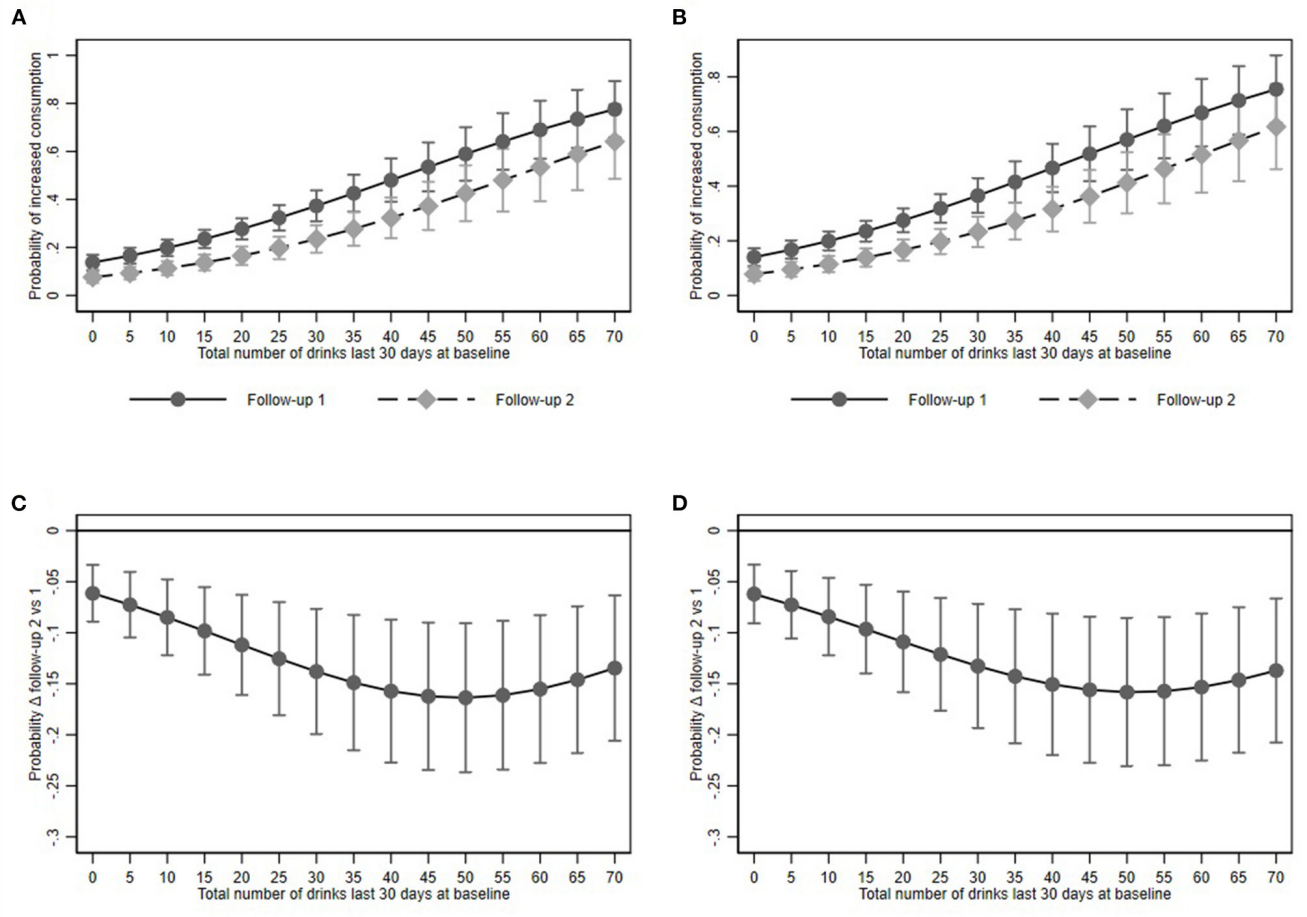
Increased alcohol consumption and its association with baseline alcohol consumption at Follow-up 1 and 2. **(A,B)** show predictive margins and **(C,D)** average marginal effects for the restricted and full models respectively. Dots represent point estimates. Whiskers show the 95% confidence interval.

Predictive margins, i.e., the predicted percentage of university students reporting increased alcohol consumption was 23.7% (95% CI: 20.3–27.1) and 14.7% (95% CI: 11.6–17.8) at the first and second follow-up, and 23.7% (95% CI: 20.3–27.2) and 14.9% (95% CI: 11.7–18.0) at the first and second follow-up in the restricted and the full model respectively (Figure 1).

### Any Binge Drinking Event

University students who consumed more alcoholic drinks at baseline were more likely to report at least one binge drinking event in the past 30 days in the restricted model (OR = 1.08, 95% CI: 1.05–1.10) and the full model (OR = 1.08, OR = 1.05–1.10). However, we did not find a statistically significant difference between the first and the second follow-up.

Men as compared to women (OR = 2.24, 95% CI: 1.40–3.60) and university students with higher anxiety scores (OR = 1.06, 95% CI: 1.01–1.12) were more likely to report binge drinking on at least one occasion. Moreover, older university students (OR = 0.94, 95% CI: 0.91–0.98) and those living in their parents' household (OR = 0.54, 95% CI: 0.33–0.87) were less likely to report any binge drinking event. Remaining covariates were not associated with binge drinking on at least one occasion.

**Figure 2:**
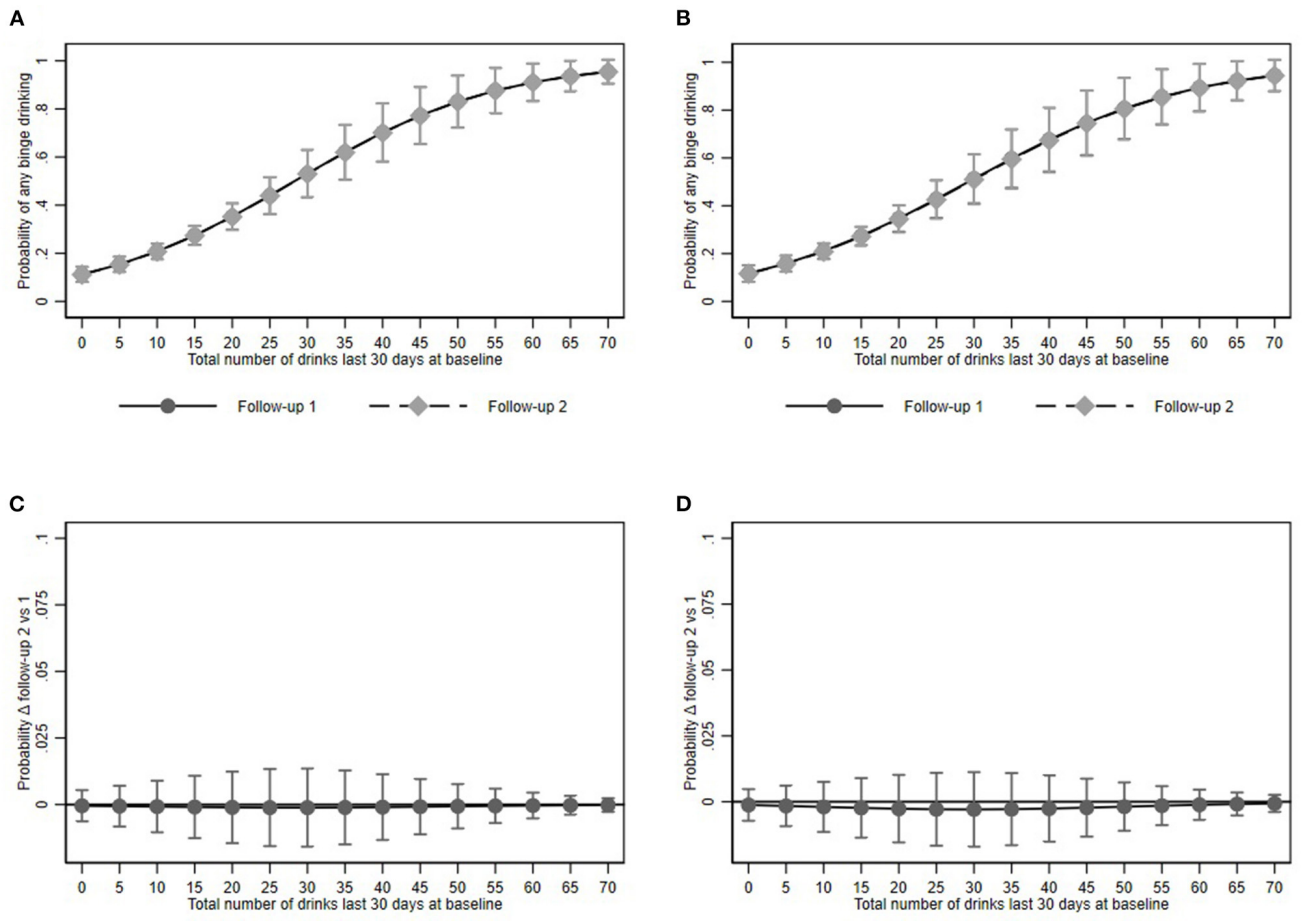
Any binge drinking episode and its association with baseline alcohol consumption at Follow-up 1 and 2. **(A,B)** show predictive margins and **(C,D)** average marginal effects for the restricted and full models respectively. Dots represent point estimates. Whiskers show the 95% confidence interval.

Predictive margins for binge drinking in the restricted model were 26.9% (95% CI: 23.9–30.0) and 26.9% (95% CI: 23.8–30.0). In the full model the predicted proportion amounted to 27.0% (95% CI: 24.0–30.0) and 26.8 (95% CI: 23.8–29.8) respectively (Figure 2).

### Heavy Binge Drinking

Similar to any binge drinking, university students who consumed more alcoholic drinks at baseline were more likely to report heavy binge drinking in the past 30 days in the restricted model (OR = 1.07, 95% CI: 1.06–1.09) and the full model (OR = 1.08, 95% CI: 1.06–1.09) and we did not find a statistically significant difference between the first and the second follow-up. With respect to covariates, university students with higher social support were more likely to report heavy binge drinking (OR = 1.28, 95% CI: 1.00–1.66). Remaining covariates were not associated with heavy binge drinking.

**Figure 3:**
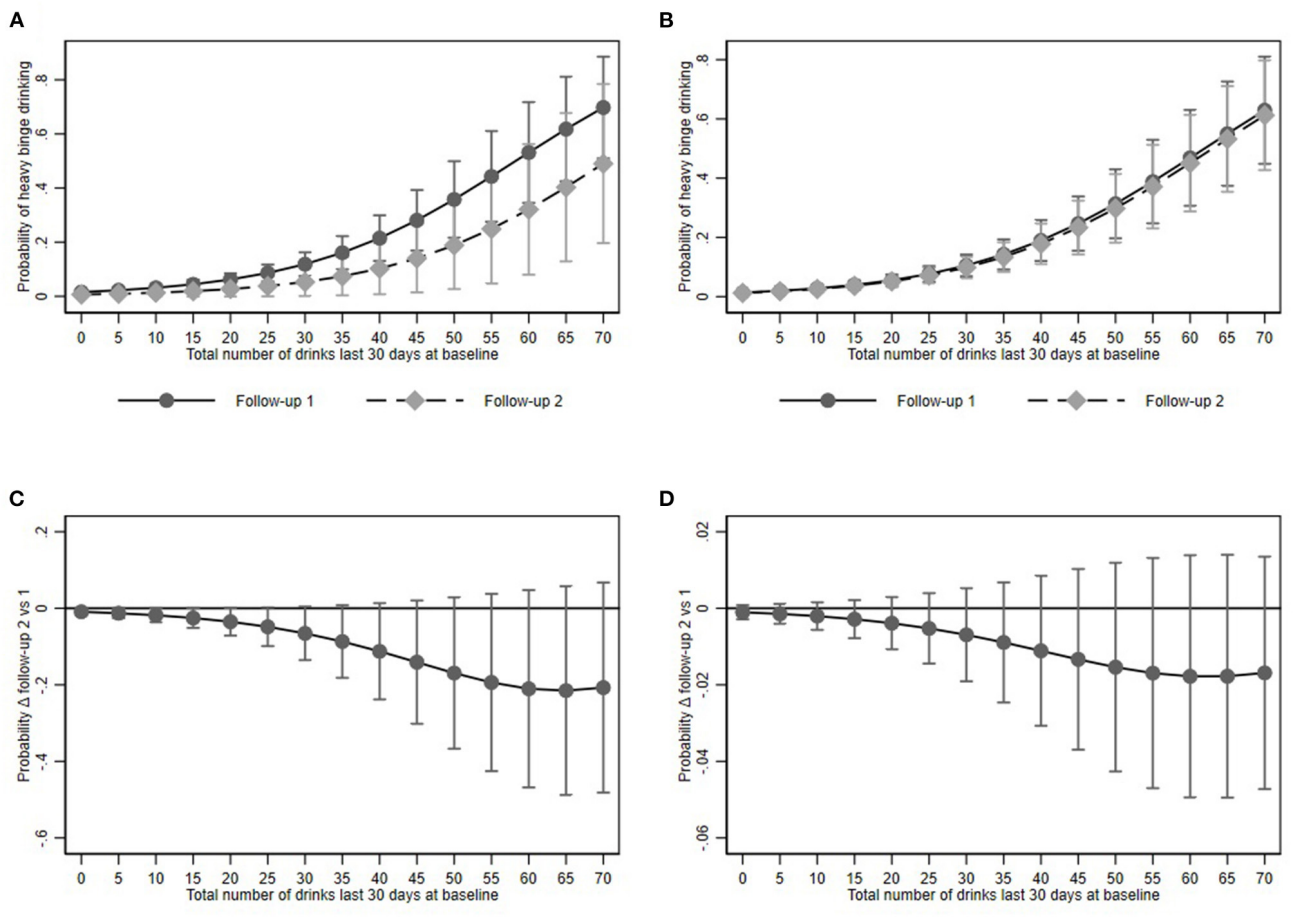
Heavy binge drinking and its association with baseline alcohol consumption at Follow-up 1 and 2. **(A,B)** show predictive margins and **(C,D)** average marginal effects for the restricted and full models respectively. Dots represent point estimates. Whiskers show the 95% confidence interval.

Predictive margins for binge drinking in the restricted model were 7.0% (95% CI: 5.2–9.0) and 3.9% (95% CI: 1.2–6.7). In the full model the predicted proportion was 6.3% (95% CI: 5.0–8.3) and 6.3% (95% CI: 4.7–7.9) respectively (Figure 3).

## Discussion

During the first months of the COVID-19 pandemic, a fifth of our sample of Swiss university students reported an increased consumption of alcohol and a quarter engaged in binge drinking. We found that the number of drinks at baseline were associated with a higher probability of increased alcohol consumption, as well as engaging in single and multiple binge drinking events. Further, higher anxiety scores were associated with a higher probability to increase alcohol consumption and engaging at least once in binge drinking. Additional factors associated with any binge drinking were male gender, younger age and not living with parents. Higher perceived social support was only associated with engaging in heavy binge drinking, but not with a higher probability to increase alcohol consumption or any binge drinking.

While the majority of university students reported no increase in alcohol consumption at the two survey time points, one in five reported an increase in alcohol consumption. This finding is comparable with other study results (5, 16), indicating differential health behavior changes among university students. University students who increased their alcohol consumption reported a higher number of drinks at baseline, higher anxiety symptoms and lower resilience compared to university students who did not increase their alcohol consumption. Apparently, university students who reported a higher alcohol consumption at the beginning of the pandemic were those at highest risk to increase their alcohol consumption during the following weeks of the early pandemic. We did not assess reasons for the increase in alcohol consumption, however, other studies suggest that university students used alcohol to cope with the insecure and challenging situation caused by the pandemic (38, 39). Vanderbruggen et al. found loneliness, lack of social contact, loss of daily structures but also boredom to be the main reasons for drinking more alcohol during the pandemic (40). The higher anxiety symptoms and lower resilience level reported by university students in the current study further support the hypothesis that the perceived adverse impact of the pandemic may have led to the increase in alcohol consumption.

The proportion of university students who increased their alcohol consumption was higher at follow-up one (24.9%) as compared to follow-up two (14.1%). This reduction might be explained by the loosening of containment measures and a less severe epidemiological situation at T2 (i.e., lower COVID-19 cases and deaths compared to T0 and T1, see Supplementary Figure 1). The first follow-up took place shortly after the lockdown, therefore we may assume that the higher proportion of students with increased alcohol consummation indicates its use as coping mechanism or a lock-down induced temporary health behavior change. At the second follow-up increased alcohol consumption dropped by 10%. This reduction might be explained by the loosening of containment measures and a less severe epidemiological situation at T2 (i.e., lower COVID-19 cases and deaths compared to T0 and T1, see Supplementary Figure 1), thus a normalization of everyday life and reductions of Covid-related stressors.

Binge drinking represents a risky health behavior, which occurs mainly at young ages during parties and/or with peers (41). In this sample, about one forth engaged at least once in binge drinking during the last 30 days at T1 or T2. The finding is comparable to results from a national Swiss survey, in which 27% of 15- to 24-year-olds and 22% of 25-to-34-year-olds engaged at least once a month in binge drinking (42). Also, for binge drinking higher number of drinks at baseline and anxiety symptoms were associated with engaging in binge drinking at follow-up. Interestingly, university students that lived with their parents showed lower odds for binge drinking than university students living in their own household. Possibly living with parents is a proxy for higher social control or, positively expressed, parents acted as a support system providing a daily structure at home and social contacts that the other university students lacked, reducing the risk to resort to binge drinking as a coping strategy. Further risk factors were male gender and younger age. Both associations were expected and shown in previous studies (5, 41).

Heavy binge drinking was not associated with higher anxiety symptoms, lower resilience or self-efficacy, which implies that the reported increase possibly was not a coping mechanism. In fact, the behavior was associated with higher perceived social support, which seems counter-intuitive at first. However, binge drinking does happen more often among friends and peers (39, 41). Perceived social support was added in the model under the assumption to be a protective factor, but in this case we observe the opposite. The results should be interpreted with caution, as the sample of heavy binge drinker was rather small, and other known associations, such as gender and age, were not found. However, heavy binge drinking in university students is a known problem and based on previous findings, it becomes clear that successful interventions must address perceived social norms among university students in relation to binge drinking (41, 43).

A limitation of the study is that self-reported data on alcohol consumption might be affected by social desirability. Social desirability bias, however, would most probably cause an underreporting; implying that the actual number of participants with increased consumption might be higher. Further, it is possible that only the first or last days of the 30-act period are remembered more accurately, while the time in between is more difficult to remember [primacy and recency effect (44, 45)]. So, an over- and underestimation of reported consumption is possible. Assuming a non-differential bias, the power but not the direction of the association should be impacted. Furthermore, we did not assess the reasons for drinking or the situations in which university students were drinking alcohol. Also, our results may not be generalizable to Swiss young adults outside the university setting, as the latter may have been affected differently by the pandemic, and the higher proportion of women compared to men could limit the generalizability of the frequencies of increased alcohol consumption. A strength of the study is the large sample of university students from different faculties and the data collection started early in the pandemic, including the first lockdown, as well as the post-lockdown phase. Furthermore, it is the first study examining risk factors for increased or binge alcohol drinking in Swiss university students during the pandemic, providing valuable information for health promotion and prevention for this specific population in future.

Our study indicates that the lockdown has had a significant impact on short-term alcohol consumption and binge drinking in university students. It is reassuring, that at the follow-up 2 the odds of increased alcohol consumption was already reduced. The data underline the vulnerability of the transition to an independent adult life (26) and the relevance of the life phase emerging adulthood for health behavior development. It seems relevant to consider risks simultaneously (46) or using a cluster approach to identify different risk profiles, not only including age and gender, but also living situation and social support when developing interventions. The data point to the importance of the baseline risk when a crisis occurs, and thus the necessity of continuous prevention activities and investments in order to increase resilience. Universities, for example, could teach sustainable coping strategies for students to deal with uncertain and stressful events and provide support for those students at risk.

The present study showed that one in four university students developed a more risky health behavior regarding alcohol consumption in the early phase of the pandemic. It is important to identify university students at risk and design target prevention including factors such as age, gender, living situation, and social norms. Further, health behavior and determinants of health behaviors of university students should be monitored carefully during the further course of the pandemic.

## Data Availability Statement

The raw data supporting the conclusions of this article will be made available by the authors, without undue reservation.

## Ethics Statement

The studies involving human participants were reviewed and approved by the local cantonal Ethics Committee (BASEC-Nr. Req-2020-00326) and the ZHAW data protection officer. The patients/participants provided their written informed consent to participate in this study.

## Author Contributions

JD, TV, and AZ: conceptualization, methodology, and investigation. TV: formal analysis and data curation. AZ: writing original draft. TV, AZ, SA, AW, and JD: review and editing. JD and TV: supervision and funding acquisition (internal funding). JD: project administration. All authors have read and agreed to the published version of the manuscript.

## Conflict of Interest

The authors declare that the research was conducted in the absence of any commercial or financial relationships that could be construed as a potential conflict of interest.

## Publisher's Note

All claims expressed in this article are solely those of the authors and do not necessarily represent those of their affiliated organizations, or those of the publisher, the editors and the reviewers. Any product that may be evaluated in this article, or claim that may be made by its manufacturer, is not guaranteed or endorsed by the publisher.
