# Supplementary material for: Change in Alcohol Consumption and Binge Drinking in University Students During the Early COVID-19 Pandemic
Source: Front Public Health. 2022 Apr 27;10:854350. doi: 10.3389/fpubh.2022.854350 (PMC9092343; doi:10.3389/fpubh.2022.854350)
Supplement: Supplementary file 1 [file Data_Sheet_1.PDF]

Supplementary Material

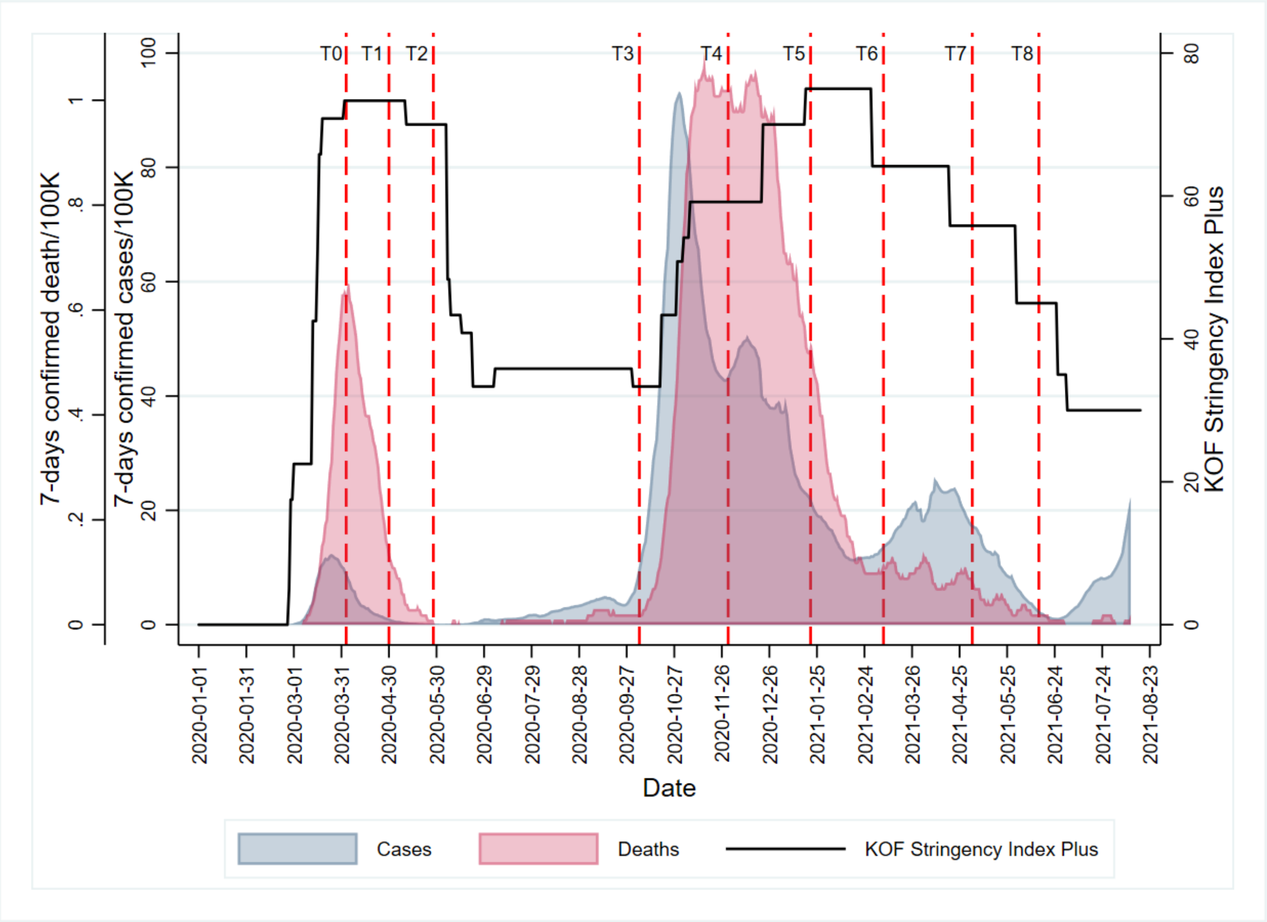

Supplementary Figure 1. The three survey time points (T0, T1, T2) are presented in relation to COVID-19 incidence, death and stringency of measures in Switzerland.

Note. The KOF Stringency Index and KOF Stringency-Plus Index document the stringency of Covid 19 measures in Switzerland.
